# Prognostic importance of IDH mutations in chondrosarcoma: An individual patient data meta‐analysis

**DOI:** 10.1002/cam4.4019

**Published:** 2021-06-03

**Authors:** Huy Gia Vuong, Tam N.M. Ngo, Ian F. Dunn

**Affiliations:** ^1^ Department of Pathology Oklahoma University Health Sciences Center Oklahoma City OK USA; ^2^ Stephenson Cancer Center Oklahoma University Health Sciences Center Oklahoma City OK USA; ^3^ Faculty of Medicine Pham Ngoc Thach University of Medicine Ho Chi Minh City Vietnam; ^4^ Department of Neurosurgery Oklahoma University Health Sciences Center Oklahoma City OK USA

**Keywords:** chondrosarcoma, IDH, isocitrate dehydrogenase, meta‐analysis, overall survival, recurrence‐free survival

## Abstract

**Introduction:**

*IDH1*/*2* mutations are prevalent in cartilaginous tumors including chondrosarcoma. This meta‐analysis using individual patient data (IPD) aimed to investigate the clinical and prognostic association of these mutations in chondrosarcoma patients.

**Methods:**

Two electronic databases including PubMed and Web of Science were searched for relevant data. We included studies providing IPD of chondrosarcoma with available *IDH1*/*2* mutational status for meta‐analysis. Chi‐square and *t*‐test were performed to compare the groups with and without *IDH1*/*2* mutations. For survival analysis, log‐rank test, and Cox proportional hazards model were used to investigate the association of *IDH* mutations with patient outcomes.

**Results:**

Fourteen studies with 488 patients were analyzed. *IDH1* and *IDH2* mutations were detected in 38.7% and 12.1% of cases, respectively. *IDH1*/*2* mutations were significantly associated with an older age (*p* = 0.003), tumor origins (*p* < 0.001), tumor grades (*p* < 0.001), larger diameter (*p* = 0.003), relapse (*p* = 0.014), and patient mortality (*p* = 0.04). Multivariate Cox regression analysis adjusted for age, gender, tumor grade, and tumor sites confirmed the negative impact of *IDH1*/*2* mutations on patient overall survival (HR = 1.90; 95% CI = 1.06–3.42; *p* = 0.03).

**Conclusion:**

Our meta‐analysis demonstrated the distinct characteristics of *IDH1*/*2*‐mutated chondrosarcomas in comparison to those without mutations. These mutations could serve as an independent prognostic biomarker to better prognosticate patient outcomes and design appropriate treatment plans.

## 1 INTRODUCTION

Chondrosarcoma is a common primary malignant bone tumor, and is the second most common malignancy following osteosarcoma.^1^ They are classified into different grades based on tumor cellularity and nuclear changes in chondrocytes. Low‐grade chondrosarcomas correspond to grade I tumors. These tumors are usually treated with curettage and wide excision and likely have a good prognosis^2^, ^3^ whereas, high‐grade (grade II–III) chondrosarcomas have a high risk of relapse and even metastasis which requires more aggressive treatment.^3^, ^4^ The most aggressive form of chondrosarcoma is dedifferentiated chondrosarcoma, which is associated with a dismal prognosis and rapid development of widespread metastases.^5^

Besides tumor grades, several clinicopathological features have been shown to be of prognostic importance in chondrosarcoma patients including gender, tumor location, diameter, and extent of resection.^6^, ^7^, ^8^ Recently, emerging data have clarified the genomic landscape of chondrosarcomas.^9^, ^10^, ^11^, ^12^ *Isocitrate* *dehydrogenase* (*IDH)* mutation is a common genetic alteration in gliomas^13^ and is also found in about 50% of central chondrosarcomas.^10^ In gliomas, this mutation is associated with a favorable prognosis.^14^, ^15^ However, published data are equivocal regarding the association of *IDH1*/*2* mutations and outcomes of chondrosarcoma patients.^12^, ^16^, ^17^ This meta‐analysis aims to explore the clinicopathological and prognostic characteristics of *IDH1*/*2* mutations in chondrosarcoma.

## 2 METHODS

### 2.1 Ethical approval

An ethical approval is not needed for this study because this is a meta‐analysis and systematic review based on published studies.

### 2.2 Literature search

PubMed and Web of Science databases were searched for relevant articles from inception to November 2020. We used the following term: chondrosarcoma AND (IDH1 OR IDH2 OR IDH1/2 OR IDH OR isocitrate dehydrogenase). This study generally followed the recommendations of the Preferred Reporting Items for Systematic Review and Meta‐Analysis (PRISMA) statement.^18^

### 2.3 Selection criteria and abstract screening

All search results from two electronic databases were imported into EndNote (Clarivate, PA, US) and duplicates were removed. Two reviewers independently screened the title and abstract of these search results. Studies were included if they provided individual patient data (IPD) of chondrosarcoma patients and *IDH1*/*2* mutation data. We excluded studies without IPD; reviews; case reports, proceeding papers, conference abstracts, or books. If there are any discrepancies among the two reviewers, discussion and consensus were reached.

### 2.4 Full‐text screening and data extraction

The full‐text of suspected studies were independently read by two reviewers and IPD were extracted into a standardized worksheet. The following IPD were extracted: authors, institution, country, year of publication, demographic information, tumor location, tumor diameter, histopathological subtypes, tumor grades, patient outcomes (recurrence, recurrence‐free survival [RFS] time, metastasis, metastasis‐free survival [MFS] time, overall survival [OS] status, OS time), and status of *IDH1*/*2* mutations.

### 2.5 Statistical analyses

Categorical data were presented as frequency (percentage), and comparisons between groups were performed using the chi‐square test. Continuous variables are expressed as mean ± standard deviation (SD) for normal distributions and median + interquartile range (IQR) for non‐normal distributions. The distribution of continuous variables was assessed using skewness, kurtosis, and visual inspection of the histogram. Continuous variables were compared between two groups by *t*‐test or Mann–Whitney *U*‐test, as appropriate. Univariate and multivariable Cox proportional hazards models were conducted to determine the association of *IDH1*/*2* mutations with clinical outcomes (recurrence, metastasis, and overall survival). Proportionality assumptions of the Cox regression models were assessed by log‐log survival curves and with the use of Schoenfeld residuals. The deviance residuals and the dfbeta values were used to examine influential observations. Hazard ratios (HR) are presented as mean and 95% confidence interval (CI). A two‐sided *p*‐value of <0.05 was considered statistically significant. The statistical analyses were performed using IBM SPSS Statistics for Windows, version 22.0 (IBM Corp., Armonk, N.Y., USA) and R software, version 3.6.1 (The R Foundation, Vienna, Austria).

### 2.6 Quality assessment and risk of bias analysis

We evaluated the quality of included studies in our meta‐analysis using the Newcastle–Ottawa Scale (NOS).^19^ Two reviewers independently awarded the number of stars using a standardized checklist. We considered studies moderate to high quality if they have six stars or more.

## 3 RESULTS

**FIGURE 1:**
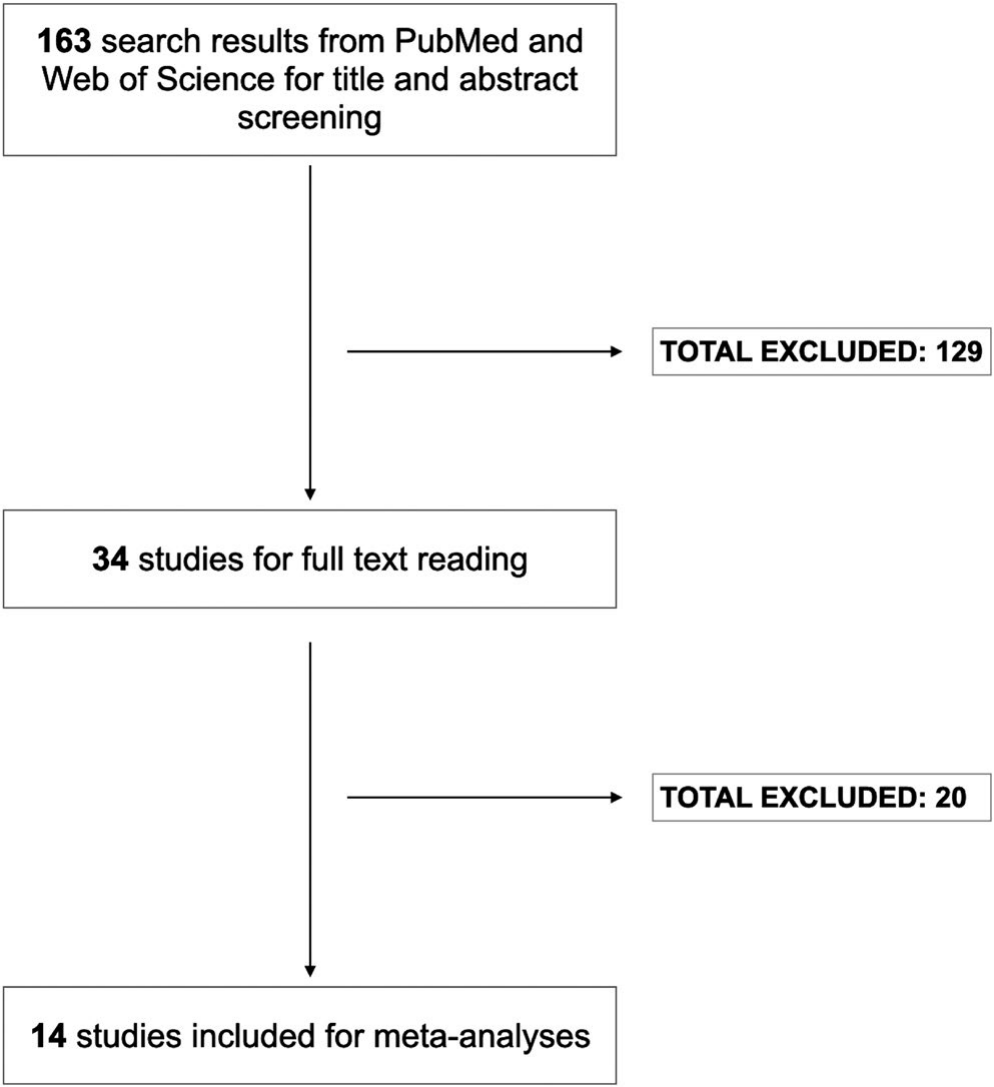
Study flowchart

We identified 136 articles for the title and abstract screening; 34 of these were selected for full‐text reading. Following this step, we included 14 studies comprising 488 chondrosarcoma patients with available *IDH1*/*2* mutational status for data analyses (Figure 1).^10^, ^11^, ^12^, ^17^, ^29^ All studies but three had moderate to good quality using the NOS tool assessment. Table 1 and Table S1 present the characteristics of all included studies.

**TABLE 1:** Characteristics of included studies

Study	Institution	Country	Study period	Detection methods	No. of cases	NOS stars		
						S	C	O
Amary (2011)^10^	Multicenter	UK	NA	Sequenom, Sanger, IHC	69	4	0	3
Arai (2012)^20^	Gunma University School of Medicine	Japan	NA	Sanger	13	4	0	1
Asioli (2020)^17^	Multicenter	Italy	1998–2019	Sanger, IHC	46	4	0	3
Chen (2017)^21^	Multicenter	USA	NA	IDH1/2 Rotor‐Gene Q PCR, IHC	43	4	0	1
Gambarotti (2020)^22^	IRCCS Instituto Ortopedico Rizzoli	Italy	1990–2020	Sanger	6	4	0	3
Kanamori (2015)^23^	Keio University Hospital	Japan	1997–2010	Sanger	7	4	0	3
Kerr (2013)^11^	Massachusetts General Hospital	USA	1990–2012	SNaPshot, Sanger	23	4	0	3
Lam (2019)^24^	Leiden University Medical Center	Netherlands	NA	NGS, Sanger	5	4	0	3
Lucas (2020)^25^	University of California at San Francisco	USA	NA	NGS, IHC	11	4	0	2
Mohammad (2020)^26^	University of British Columbia	Canada	1996–2016	Quantitative PCR, Sanger	21	4	0	3
Nicolle (2019)^27^	Multicenter	France	1997–2013	NGS	76	4	0	3
Tallegas (2019)^28^	French Bone Pathology Group Network RESOS	France	2000–2018	Pyrosequencing, Sanger	71	4	0	1
Yang (2020)^29^	Shanghai Jiaotong University Affiliated Sixth People's Hospital	China	2011–2017	Sanger	18	4	0	3
Zhu (2020)^12^	Memorial Sloan Kettering Cancer Center	USA	1984–2017	Sequenom, NGS, Sanger	79	4	0	3

Abbreviations: C, comparability; IHC, immunohistochemistry; NA, not available; NGS, next‐generation sequencing; NOS, Newcastle‐Ottawa Scale; O, outcome; PCR, polymerase chain reaction; S, selection.

### 3.1 Prevalence of *IDH* mutations in chondrosarcomas

*IDH1*/*2* mutations were detected in 250 patients (51.2%). The prevalence of *IDH1* and *IDH2* were 38.7% and 12.1%, respectively. *IDH1* and *IDH2* mutations were mutually exclusive except for one case. Among 488 analyzed cases, *IDH1* and *IDH2* mutation genotypes were reported in 237 cases. The detailed genotypes of *IDH1* and *IDH2* mutations are shown in Table 2. The prevalence of *IDH* mutations was statistically different between tumor locations with the highest prevalence seen in phalanges (100%) and femur (83.0%) and the lowest prevalence found in vertebrae and sternum (0%) (Table 3).

**TABLE 2:** Distribution of *IDH* genotypes in chondrosarcomas

*IDH* gene	*IDH* genotypes	No. of positive cases (%)
*IDH1*	R132, unspecified	13 (5.5)
	R132C	81(34.2)
	R132F	1(0.4)
	R132G	30 (12.7)
	R132H	17 (7.2)
	R132I	1 (0.4)
	R132L	24 (10.1)
	R132S	13 (5.5)
*IDH2*	R172, unspecified	11 (4.6)
	R172G	4 (1.7)
	R172 M	6 (2.5)
	R172S	31 (13.1)
	R172T	2 (0.8)
	T172W	1 (0.4)
	Others	2 (0.8)
	Total	237

**TABLE 3:** The associations of *IDH* mutations with clinicopathological factors and prognostic outcome

Variables	Groups	IDH1/2‐mut	IDH1/2‐wt	*p*‐value
Age (year)	Mean ±SD	57.2 ± 15.3	52.0 ± 16.2	**0.003**
Gender	Female	66 (37.3)	72 (43.9)	0.21
	Male	111 (62.7)	92 (56.1)	
Tumor origins	Extraosseous	5 (2.4)	53 (25.6)	**<0.001**
	Flat bone	95 (46.3)	95 (43.3)	
	Irregular bone	0 (0)	14 (9.0)	
	Long bone	104 (50.7)	39 (18.8)	
	Multiple sites	1 (0.5)	6 (2.9)	
Tumor sites	Cranium	44 (21.5)	52 (25.1)	**<0.001**
	Tracheolarynx	4 (2.0)	41 (19.8)	
	Scapula	8 (3.9)	5 (2.4)	
	Humerus	20 (9.8)	18 (8.7)	
	Chest wall (rib & sternum)	4 (2.0)	19 (9.2)	
	Vertebra	0 (0.0)	14 (6.8)	
	Pelvis	40 (19.5)	29 (14.0)	
	Femur	73 (35.6)	15 (7.2)	
	Fibula/Tibia	7 (3.4)	6 (2.9)	
	Phalanges	4 (2.0)	0 (0.0)	
	Others	1 (0.5)	8 (3.8)	
Tumor grades	I	24 (10.0)	54 (23.8)	**<0.001**
	II	103 (43.1)	124 (54.6)	
	III	24 (10.0)	13 (5.7)	
	Dedifferentiated	88 (36.8)	36 (15.9)	
Largest diameter (cm)	Mean ±SD	12.1 ± 7.2	9.5 ± 6.7	**0.003**
Recurrence	Yes	12 (31.6)	19 (61.3)	**0.014**
	No	26 (68.4)	12 (38.7)	
Metastasis	Yes	52 (54.7)	42 (55.3)	0.95
	No	43 (45.3)	34 (44.7)	
Death	Yes	64 (47.8)	26 (33.3)	**0.04**
	No	70 (52.2)	52 (66.7)	

Abbreviations: HG‐CCS, high‐grade chondrosarcoma; IQR, interquartile range; LG‐CCS, low‐grade chondrosarcoma; SD, standard deviation.

Bold values indicate a statistically significant result.

### 3.2 Associations of *IDH* mutations with clinicopathological factors and patient outcomes

Table 3 shows the correlations of *IDH* mutations with clinicopathological features. Compared to *IDH*‐wild type (*IDH*‐wt) tumors, *IDH*‐mut chondrosarcomas were associated with older age and larger tumor diameter (*p* = 0.003). In addition, *IDH* mutations were more likely to occur in long bones chondrosarcomas (e.g., femur, humerus, tibia) and flat bones (e.g., pelvis, cranium) whereas, these mutations were completely absent in irregular bones (e.g., vertebrae, sternum). We also found an association of *IDH* mutations with chondrosarcoma grades; the prevalence of *IDH* mutations was significantly increased with grades with the highest prevalence being identified in dedifferentiated tumors.

**FIGURE 2:**
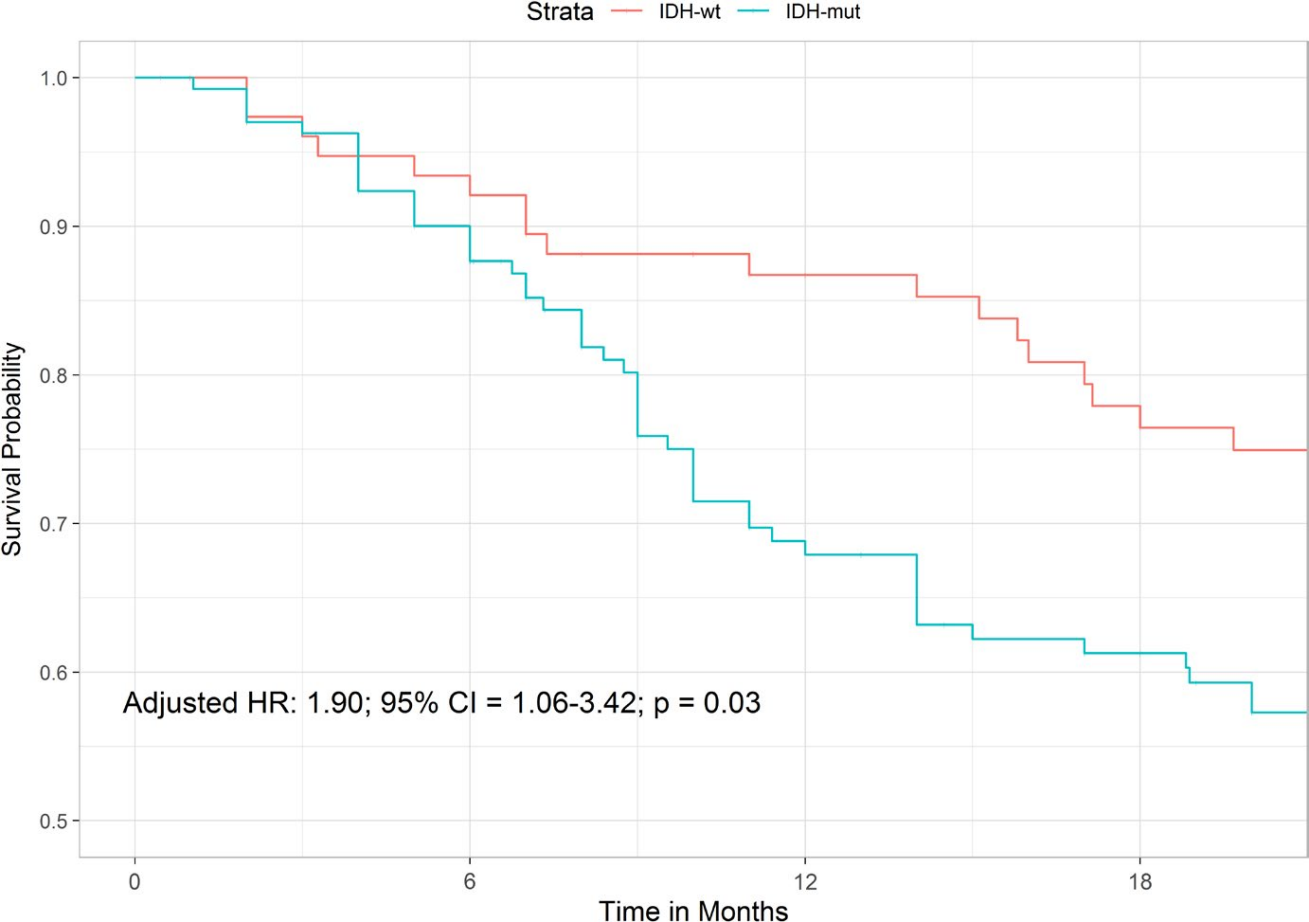
Kaplan–Meier curve illustrating the impact of *IDH* mutations on OS of chondrosarcoma

The median follow‐up duration of chondrosarcomas was 20.5 months. The presence of *IDH1*/*2* mutations were associated with a decreased recurrence rate but a significantly higher risk of patient death (Table 3). For time‐to‐event analysis, *IDH1*/*2* mutations were not associated with tumor RFS (HR = 0.63; 95% CI = 0.30–1.31; *p* = 0.21) and MFS (HR = 1.75; 95% CI = 0.91–3.36; *p* = 0.09), but they were significantly correlated with patient OS (HR = 1.81; 95% CI = 1.15–2.87; *p* = 0.01) (Figure 2). The significant result was retained in the multivariate model adjusted for age, gender, tumor grades, and tumor locations (HR = 1.90; 95% CI = 1.06–3.42; *p* = 0.03).

**FIGURE 3:**
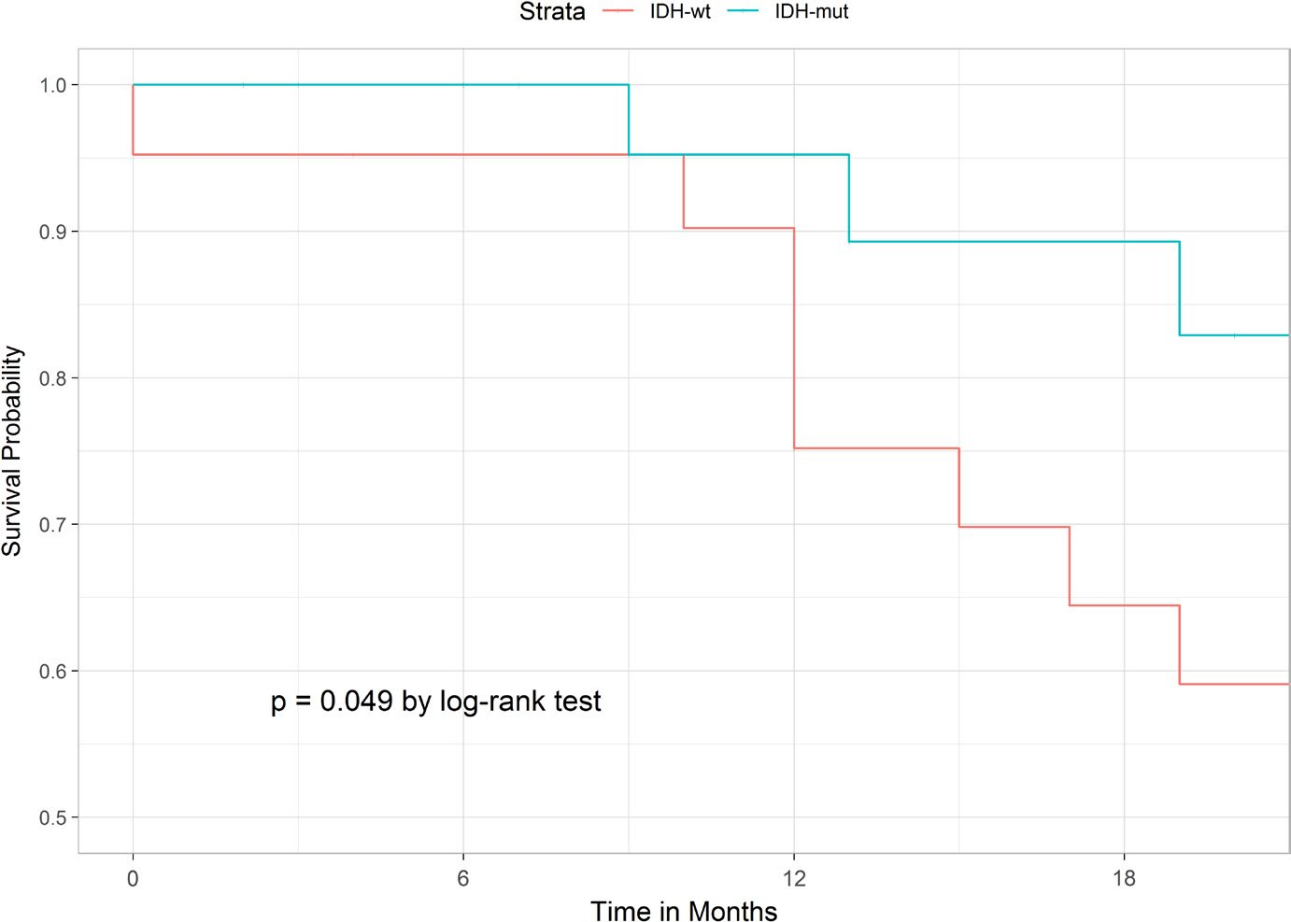
Kaplan–Meier curve illustrating the impact of *IDH* mutations on RFS of high‐grade chondrosarcoma

Stratified by tumor grades, we found an association of *IDH* mutation with longer RFS in the high‐grade group (Figure 3) using the log‐rank test (*p* = 0.049). Because of the small sample size with available survival data when dividing into different subgroups, we did not found any associations of *IDH1*/*2* mutations with patient RFS, MFS, and OS using the Cox proportional hazards model in other chondrosarcoma grades (Table 4).

**TABLE 4:** Subgroup analyses of impacts of *IDH* mutations on patient outcomes in different chondrosarcoma grades

Tumor grades	Hazard Ratio (95% CI)	*p*‐value
Low‐grade		
RFS	NA	NA
MFS	NA	NA
OS	NA	NA
High‐grade		
RFS	0.41 (95% CI = 0.17–1.03)	0.06
MFS	1.55 (95% CI = 0.57–4.24)	0.39
OS	1.21 (95% CI = 0.53–2.79)	0.65
Dedifferentiated		
RFS	1.54 (95% CI = 0.25–9.27)	0.64
MFS	1.35 (95% CI = 0.54–3.34)	0.52
OS	1.63 (95% CI = 0.89–3.01)	0.12

Abbreviations: CI, confidence interval; MFS, metastasis‐free survival; NA, not available; OS, overall survival; RFS, recurrence‐free survival.

### 3.3 The characteristics of cranial chondrosarcomas

There were 96 cases of cranial chondrosarcoma included in our meta‐analysis. The characteristics of these neoplasms are summarized in Table 5. *IDH* mutations were only detected in skull base chondrosarcoma and were not present in craniofacial tumors (*p* < 0.001). Patient outcomes were missing in the majority of cranial chondrosarcomas so we excluded them from the analyses.

**TABLE 5:** Characteristics of cranial chondrosarcomas

Variables	Groups	Craniofacial (%) (*n* = 23)	Skull base (%) (*n* = 73)	*p*‐value
Gender	Male	6 (42.9)	17 (37.8)	0.734
	Female	8 (57.1)	28 (62.2)	
Age	Mean ±SD	57.5 ± 19.7	49.9 ± 15.4	0.135
Grade	I	8 (36.4)	20 (30.8)	0.160
	II	13 (59.1)	44 (67.7)	
	III	0 (0.0)	1 (1.5)	
	Dedifferentiated	1 (4.5)	0 (0.0)	
*IDH1*/*2* status	Mutated	0 (0.0)	44 (60.3)	**<0.001**
	Wild‐type	23 (100)	29 (39.7)	

Abbreviations: SD, standard deviation.

Bold values indicate a statistically significant result.

## 4 DISCUSSION

*IDH* mutation is a highly recurrent genetic event in glial and hematopoietic tumors.^13^, ^30^ In mice, there is established evidence that mutant *IDH* can trigger enchondromatosis, which could subsequently undergo additional tumorigenesis to become chondrosarcoma.^31^ Our study confirmed the presence of this mutation in over 50% of chondrosarcoma patients and its prevalence is elevated with tumor grade with the highest prevalence seen in dedifferentiated tumors. *IDH* mutations are very specific to cartilaginous tumors but do not exist in other mesenchymal neoplasms such as osteosarcoma or undifferentiated pleomorphic sarcoma.^11^, ^21^, ^29^ Of note, it is crucial to distinguish chondrosarcoma from osteosarcoma because the former is not chemosensitive while the primary treatment of osteosarcoma involves neoadjuvant chemotherapy.^32^ Additionally, about a quarter of conventional osteosarcoma can produce cartilaginous areas, making it challenging to distinguish these two tumor entities in small biopsies. Unlike gliomas where *IDH1* R132H mutation is predominant and can be diagnosed by immunohistochemistry,^33^ the distribution of *IDH1* mutations is more heterogeneous in chondrosarcoma with R132C being the most frequent followed by R132G and R132L. As there are no antibodies specific for R132C currently available, the best approach to detect this hotspot mutation is to use traditional Sanger sequencing or pyrosequencing. *IDH2* mutations are prevalent in patients with acute myeloid leukemia and occur most frequently at codon 140.^13^ We did not find any *IDH2* R140 mutations among the included studies. *IDH2* mutation solely occurred at codon 172 in chondrosarcomas with R172S being the most frequent *IDH2* genotype.

Our results established a strikingly heterogeneous distribution of *IDH1*/*2* mutations in specific anatomical locations. The distribution of *IDH*‐positive tumors in the bones of extremities was significantly higher than other types, suggesting different pathways of tumorigenesis among these neoplasms. Besides *IDH* mutation, other common genetic alterations in chondrosarcoma include *COL2A1*, *CDKN2A*/*B*, *TP53* mutations with a prevalence of 20–30%.^12^, ^27^ *TERT* promoter mutation/amplification was found in a subset of high‐grade chondrosarcomas and likely to concurrently occur with *IDH* mutations, *CDKN2A*/*B* deletions, or *TP53* mutations.^12^ That the effect of the *TERT* promoter mutation on patient survival can be modulated by *IDH* mutation and other genetic events in glioma patients has been suggested in prior studies.^34^, ^35^, ^36^

In cranial chondrosarcomas, we demonstrated different patterns of *IDH1*/*2* mutations by the anatomical site of neoplasms. These mutations were found in 60% of skull base tumors but did not exist in craniofacial chondrosarcomas. These discrepancies might stem from the distinct modes of ossification where skull base bones are structured by endochondral ossification and facial bones originate from intramembranous ossification. The latter mode involves direct differentiation of progenitor cells into osteoblasts and the cartilaginous phase does not take place. The high frequency of *IDH1*/*2* mutations in skull base chondrosarcoma makes it a useful diagnostic tool to differentiate from chordoma, particularly in small biopsy specimens. The effect of *IDH* mutations on patient outcome in this group is limited and most of the included studies did not provide survival data. Kanamori et al.^23^ reported an insignificant correlation of *IDH1* mutation with tumor relapse but only seven patients were enrolled in their study. Additional studies are necessary to elucidate the prognostication of *IDH1*/*2* mutations in chondrosarcomas of the head and neck.

The impact of *IDH1*/*2* mutations on chondrosarcoma patient survival is unclear.^12^, ^16^, ^17^ Lugowska et al. indicated an association of *IDH* mutation with a shorter OS while other studies failed to establish this relationship.^12^, ^26^ This study concluded a negative impact of *IDH* mutation on patient OS, emphasizing the independent role of *IDH* mutation as a prognostic marker in chondrosarcomas. In this study, we found that *IDH* mutation is more commonly seen in dedifferentiated tumors which could explain the link between *IDH* mutation and mortality rate. It can help clinicians better predict the clinical course and tailor appropriate treatment decisions. The association of *IDH* mutation and tumor relapse remains investigational. *IDH* mutation was found to be associated with longer RFS in grade II‐III chondrosarcomas.^12^ We also observed the same trend of correlation of *IDH* mutation and patient RFS in chondrosarcomas and a decreased risk of tumor relapse in patients harboring *IDH1*/*2* mutations. Although the Cox regression model does not reach statistical significance, it should be noted that the length of follow‐up in the Zhu et al. study^12^ was quite long compared to other studies (over 21 years), increasing the power of their statistical adjustment. Raw patient survival data was not provided in this study so we could not include them in our survival analyses.

### 4.1 Strengths and limitations

Our study is the first meta‐analysis investigating the clinical and prognostic significance of *IDH* mutations in chondrosarcomas. Our meta‐analyses were solely based on individual participant data which significantly increases the statistical power compared to meta‐analyses using aggregate data. Given the rarity of chondrosarcomas,^8^ it was difficult to reach statistical significance when examining the prognosis of this tumor. Most of the published data failed to demonstrate the prognostic impact of *IDH* mutations in chondrosarcomas.^17^, ^23^, ^26^ We incorporated nearly 500 cases of chondrosarcoma and highlighted that *IDH* mutation is an independent prognostic marker regardless of age, gender, tumor grades, and locations. However, there are a few limitations that need to be addressed. First, all included studies were designed retrospectively, resulting in unavoidable selection bias. Next, few studies only selected a specific grade or type of chondrosarcoma (e.g., dedifferentiated) which can affect the true frequency of *IDH* mutations. We were unable to calculate the survival of *IDH*‐mut and *IDH*‐wt low‐grade chondrosarcomas and cranial chondrosarcomas because of the small sample size and lack of follow‐up data among the included studies. Future meta‐analyses are needed to investigate the prognostic value of *IDH1*/*2* mutations in these neoplasms. Finally, the median follow‐up time of chondrosarcomas in our series was only 20.5 months which could affect the long‐term survival analysis.

In summary, we demonstrated the distinct characteristics of chondrosarcoma patients harboring *IDH1*/*2* mutations as compared with patients without these mutations. *IDH1*/*2* mutations were prevalent in some specific types of chondrosarcoma and assessment of these mutations in challenging cases could help distinguish from chordoma or osteosarcoma. These mutations could be used as an independent prognostic marker to better predict patient outcomes.

## CONFLICTS OF INTEREST

The authors declare no conflicts of interest.

## ETHICAL APPROVAL

Not applicable.

## Supporting information

Table S1

## Data Availability

Not applicable.
