# Supplementary material for: Prognostic importance of IDH mutations in chondrosarcoma: An individual patient data meta‐analysis
Source: Cancer Med. 2021 Jun 3;10(13):4415–23. doi: 10.1002/cam4.4019 (PMC8267117; doi:10.1002/cam4.4019)
Supplement: Supplementary file 1 — Table S1 [file CAM4-10-4415-s001.docx]

**PROGNOSTIC IMPORTANCE OF IDH MUTATIONS IN CHONDROSARCOMA: AN INDIVIDUAL PATIENT DATA META-ANALYSIS**

**Table S1. Reported data among the included studies**

| Study | Gender | Age | Tumor  site | Tumor grade | Tumor size | RFS status | RFS time | MFS  status | MFS  time | OS  status | OS  time | IDH genotype |
| --- | --- | --- | --- | --- | --- | --- | --- | --- | --- | --- | --- | --- |
| Amary 2011 | Y | Y | Y | Y | Y | Y | Y | Y | Y | Y | Y | Y |
| Arai 2012 | Y | Y | Y | N | N | N | N | N | N | N | N | Y |
| Asioli 2020 | Y | Y | Y | Y | Y | N | N | N | N | N | N | Y |
| Chen 2017 | Y | Y | Y | Y | Y | N | N | N | N | N | N | Y |
| Gambarotti 2020 | Y | Y | Y | Y | Y | Y | Y | Y | Y | Y | Y | Y |
| Kanamori 2015 | Y | Y | Y | Y | N | Y | Y | N | N | Y | Y | Y |
| Kerr 2013 | Y | Y | Y | Y | Y | N | N | N | N | Y | Y | N |
| Lam 2019 | Y | Y | Y | N | Y | N | N | N | N | Y | Y | Y |
| Lucas 2020 | Y | Y | Y | Y | Y | N | N | Y | N | Y | Y | Y |
| Mohammad 2020 | Y | Y | Y | Y | Y | N | N | Y | Y | Y | Y | Y |
| Nicolle 2019 | N | N | N | Y | N | N | N | N | N | Y | Y | Y |
| Tallegas 2019 | N | N | Y | Y | N | N | N | N | N | N | N | Y |
| Yang 2020 | Y | Y | Y | Y | N | N | N | N | N | Y | Y | Y |
| Zhu 2020 | Y | Y | Y | Y | Y | N | N | Y | N | N | N | Y |

Abbreviations: MFS, metastasis-free survival; OS, overall survival; RFS, recurrence-free survival

Y indicates that variables were reported in the corresponding study

N indicates that variables were not reported in the corresponding study
